# CARF-HAD phosphatase effectors provide immunity during the type III-A CRISPR–Cas response

**DOI:** 10.1093/nar/gkaf1363

**Published:** 2025-12-19

**Authors:** Gianna Stella, Linzhi Ye, Sean F Brady, Luciano Marraffini

**Affiliations:** Laboratory of Bacteriology, The Rockefeller University, New York, NY 10065, United States; Tri-Institutional PhD Program in Chemical Biology, Weill Cornell Medical College, Rockefeller University and Memorial Sloan Kettering Cancer Center, New York, NY 10065, United States; Tri-Institutional PhD Program in Chemical Biology, Weill Cornell Medical College, Rockefeller University and Memorial Sloan Kettering Cancer Center, New York, NY 10065, United States; Laboratory of Genetically Encoded Small Molecules, The Rockefeller University, New York, NY 10065, United States; Laboratory of Genetically Encoded Small Molecules, The Rockefeller University, New York, NY 10065, United States; Laboratory of Bacteriology, The Rockefeller University, New York, NY 10065, United States; Howard Hughes Medical Institute, The Rockefeller University, New York, NY 10065, United States

## Abstract

Clustered regularly interspaced short palindromic repeats (CRISPR)–Cas (CRISPR-associated) systems provide adaptive immunity against phage infection in prokaryotes using an RNA-guided complex that recognizes complementary foreign nucleic acids. Different types of CRISPR–Cas systems have been identified that differ in their mechanism of defense. Upon infection, type III CRISPR–Cas systems employ the Cas10 complex to find phage transcripts and synthesize cyclic oligo-adenylate (cOA) messengers. These ligands bind and activate CARF immune effectors that cause cell toxicity to prevent the completion of the viral lytic cycle. Here, we investigated two proteins containing an N-terminal haloacid dehalogenase (HAD) phosphatase domain followed by four predicted transmembrane helices and a C-terminal CARF domain. We named these proteins Chp for CRISPR-associated HAD phosphatase. We show that, *in vivo*, Chp localizes to the bacterial membrane and that its activation induces a growth arrest, leads to a depletion of ATP and IMP, and prevents phage propagation during the type III CRISPR–Cas response. *In vitro*, the CARF domain of Chp binds cyclic tetra-adenylates and the HAD phosphatase domain dephosphorylates dATP, ATP, and IMP. Our findings extend the range of molecular mechanisms employed by CARF effectors to defend prokaryotes against phage infection.

## Graphical Abstract

**Graphical Abstract:**
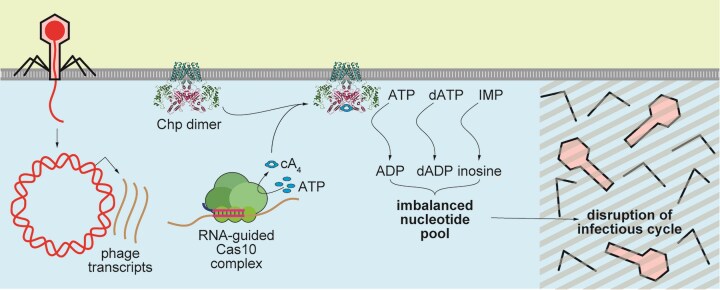

## Introduction

Prokaryotes have evolved multiple defense strategies to fight invading genetic elements such as plasmids and bacteriophages [1]. Clustered regularly interspaced short palindromic repeats (CRISPR) loci, and CRISPR-associated (Cas) genes provide RNA-guided, adaptive immunity to bacteria and archaea [2]. Depending on the *cas* gene content, CRISPR–Cas systems can be classified into different types [3]. Type III systems employ the multi-protein Cas10 complex, which harbors an RNA guide known as the CRISPR RNA (crRNA) [4]. Upon infection, annealing of the crRNA with a complementary RNA sequence present in a transcript produced by the invader [5] leads to the stimulation of three distinct activities of the Cas10 complex: (i) non-specific single-stranded DNA (ssDNA) degradation by the Cas10 HD nuclease domain [6], (ii) conversion of ATP into cyclic-oligoadenylate (cOA) second messengers by the Cas10 Palm cyclase domain [7, 8], and (iii) cleavage of the complementary target RNA [5]. Cleavage of ssDNA intermediates, which are generated during phage and plasmid DNA transcription and replication, is thought to directly destroy the invader’s genome [9]. During phage infection, this activity mediates effective immunity when the target transcript is expressed early during the lytic cycle [9, 10], presumably due to the presence of a low number of phage genomes. Synthesis of cOAs, primarily cyclic tetra- and hexa-adenylates (cA_4_ and cA_6_), activates type III immune effectors that contain a CRISPR-associated Rossman Fold (CARF) domain, which binds these second messengers [7, 8]. These effectors usually harbor a second domain whose enzymatic activity is stimulated by the binding of the cOA ligand and disrupts the host metabolism to interfere with the completion of the phage lytic cycle and limit viral spread [4]. The activation of CARF effectors is fundamental to providing immunity to the bacterial population when the target transcript is expressed late in the lytic cycle and the ssDNase activity of the Cas10 complex is not sufficient to eliminate the phage DNA from the host [9, 11–13], and results in a growth arrest of the infected cells [11–14]. Finally, cleavage of the target transcript ends both of these activities [5, 6], temporarily stopping the type III CRISPR–Cas response until another intact target transcript is recognized by the crRNA of the Cas10 complex.

Type III CRISPR loci encode many different CARF effectors, with a remarkable functional and structural diversity [4, 15–17]. To date, the activities of CARF effectors include ssDNA and ssRNA degradation [11, 18–21], membrane depolarization [12], ATP deamination [13, 22], and NAD^+^ cleavage [20]. Here, we investigated the role of a CARF effector containing a haloacid dehalogenase (HAD) phosphatase domain previously identified by a bioinformatic search of novel genes associated with CRISPR loci [23]. HAD phosphatases belong to the HAD hydrolase superfamily, one of the largest known enzyme classes, present in all three kingdoms of life [24, 25]. HAD superfamily members contain a highly conserved α/β core domain with four conserved motifs (I–IV), and in many cases also a cap domain that acts as an active site lid to sterically constrain the size and shape of its substrates [26–28]. HAD phosphatases catalyze the cleavage of P-OP bonds [29], but due to the relatively low sequence conservation across different enzymes, predicting substrate specificity is challenging. For example, one study that probed *in vitro* the substrate specificity of 23 soluble HAD phosphatases encoded in the *Escherichia coli* genome against 80 phosphorylated metabolites revealed varying degrees of substrate promiscuity [30]. The most common substrates were phosphorylated carbohydrates, pyridoxal 5′-phosphate (PLP), flavin mononucleotide (FMN), and nucleotides. Among HAD phosphatases that hydrolyzed nucleotides, YrfG preferentially hydrolyzed purine nucleotides (GMP and IMP), YjjG hydrolyzed pyrimidine nucleotides (UMP, dUMP, and dTMP) and YieH hydrolyzed both purine and pyrimidine nucleotides, although its primary substrate is glucose 6-phosphate [30]. *In vivo*, YjjG has been determined to function as a housekeeping nucleotidase that recognizes noncanonical nucleobase derivatives and prevents their misincorporation into DNA [31]. Another characterized HAD phosphatase, NagD, showed activity against UMP and GMP [32]. Outside of prokaryotic organisms, HAD phosphatases hydrolyze nucleotides in both plants [33] and mammals [34, 35]. The cellular function of many HAD hydrolases remains unknown, and it was proposed that their broad substrate specificity may serve as a reservoir for evolutionary novelty [30]. We studied the role of the CARF-HAD phosphatase effectors, which we named *Bb*Chp and *Ps*Chp, in the type III-A CRISPR–Cas immune response of staphylococci. We found that they localized to the staphylococcal membrane and, when activated by cA_4_, induced a growth arrest that protects bacteria against phage infection, most likely through the hydrolysis of ATP and dATP.

## Materials and methods

### Sequence alignments

Alignments and calculations of sequence identity and similarity were determined using either MUSCLE [36] or VectorBuilder. Alignments for Fig. 4 were manually corrected to ensure conserved residues were aligned.

### Bacterial growth

*Staphylococcus aureus* strain RN4220 [37] was grown at 37°C in brain heart infusion (BHI) medium supplemented with 10 μg mL^−1^ chloramphenicol to maintain pCRISPR and 10 μg mL^−1^ erythromycin to maintain pTarget. 5 μM CaCl_2_ was supplemented in phage experiments unless indicated otherwise.

### Plasmid cloning

The plasmids and oligonucleotides used in this study are detailed in Supplementary File S1. The amino acid sequences of *Bb*Chp and *Ps*Chp were sourced from NCBI GenBank (contigs CADBDX010000006 from *Bacteroidales* bacterium and JAGAGT010000026 from *Prevotella* sp., respectively) and synthesized by Azenta.

### *Bb*Chp toxicity assay

To measure the effect of *Bb*Chp activity on *S. aureus* growth over time, colonies of *S. aureus* containing pTarget and the specified pCRISPR were launched in liquid culture overnight in triplicate. The next day, cells were diluted 1:100 and then normalized for optical density at a wavelength of 600 nm (OD_600_) after a 1 h and 15 min outgrowth. Next, pTarget transcription was induced using 12.5 ng mL^−1^ aTc. OD_600_ readings were obtained every 10 min with a microplate reader.

### Plaque assays

Overnight cultures were launched from single colonies in 5 mL of BHI supplemented with 10 μg ml^−1^ chloramphenicol. Top agar lawns of *S. aureus* were prepared by mixing 100 μL of overnight culture with 6 ml of BHI top agar supplemented with 10 μg ml^−1^ chloramphenicol and 5 μM CaCl_2_. Top agar mixtures were poured over BHI agar plates and dried at room temperature. Serial dilutions of phage stock were prepared and spotted into top agar. Plates were incubated overnight at 37°C.

### Quantification of phage plaques

To quantify plaque-forming units (PFU) over time from cultures infected with phage, *S. aureus* cultures containing various pCRISPRs were launched overnight, diluted 1:100, and outgrown for ~1 h. Then, cells were infected with phage ΦNM1γ6 at an MOI of 1. Aliquots were taken from infected cells at the beginning of infection, 1 h, and 3 h post-infection. These aliquots were spun down to remove cells, subject to 10-fold serial dilutions, and spotted onto RN4220.

### Time-course fluorescence microscopy of phage-infected cultures

To visualize the dynamics of phage infection and immunity provided by *Bb*Chp, colonies of *S. aureus* containing various pCRISPRs with spacers programmed to target ϕNM1γ6-GFP were observed under phase contrast and in a GFP fluorescence channel as previously described [12, 13].

### Cell fractionation and western blotting

To determine localization of *Bb*Chp and *Ps*Chp, cultures were fractionated and subjected to western blotting. RN4220 cultures containing hexahistidine-tagged Csm6, Cam1, *Bb*Chp, or *Ps*Chp were launched overnight. All overnight cultures were diluted to OD_600_ of 0.05 and outgrown for 2 h. Cells were spun down at 3 900 rpm, decanted, and resuspended in lysis buffer (20 mM HEPES pH 7.1, 150 mM NaCl, 10% glycerol). Resuspended cultures were incubated with 2 mg/mL lysostaphin and a protease inhibitor cocktail, cOmplete Tablets EDTA-free EASYpack (Roche), at 37°C for 15 min. Lysates were then sonicated and spun down at 3900 rpm. An aliquot of the supernatant was collected as a whole cell lysate sample. The remaining supernatant was then subjected to ultracentrifugation at 100 000 × *g* for 1 h using a TLA-120.2 rotor. An aliquot of the supernatants was collected as a cytosolic sample and the remainder was discarded. The membrane pellets were resuspended in resuspension buffer and homogenized using a Teflon Douce homogenizer. The homogenized pellets were then subjected to another round of ultracentrifugation at 100 000 × *g* for 1 h. The supernatants were removed, and the pellets were resuspended once more in resuspension buffer using a Teflon Douce homogenizer. An aliquot was saved as a membrane sample. These samples were then run on 4%–20% Mini-PROTEAN TGX Precast Protein Gels (Bio-Rad). Transferred proteins were then probed with THE™ His Tag Antibody, mAb, Mouse (1:5000 dilution, GenScript A00186). Goat anti-mouse IgG (H + L) secondary antibody, HRP (1:10 000 dilution, Thermo Fisher 31430), was used to prepare the blot for imaging.

### Protein expression and purification

An N-terminal 6xHis-tagged *Bb*Chp CARF domain (307–528), an N-terminal 6xHis-tagged *Ps*Chp HAD domain (1–138), and an N-terminal 6xHis-tagged *Ps*Chp CARF domain (295–523) were independently cloned into a pET28 vector under an IPTG-inducible promoter and transformed into *E. coli* strain BL21 (DE3). Bacteria were grown at 37°C to an OD_600_ of 0.5–0.7 and induced with 0.5 mM isopropyl β-d-1-thiogalactopyranoside (IPTG) at 18°C overnight. Bacterial cells were spun down, resuspended in lysis buffer (20 mM HEPES pH 7.3, 400 mM NaCl, 30 mM imidazole, 10% glycerol) supplemented with protease inhibitor cocktail, cOmplete Tablets EDTA-free EASYpack (Roche), and sonicated. Cell lysates were centrifuged at 11 000 rpm for 10 min. Supernatants were filtered using a 0.22 mm PES filter and applied to a gravity column with pre-equilibrated nickel resin. The column was washed with wash buffer 1 (20 mM HEPES pH 7.3, 1 M NaCl, 30 mM imidazole, 10% glycerol), wash buffer 2 (same as lysis buffer), and then protein was eluted with elution buffer (20 mM HEPES pH 7.3, 400 mM NaCl, 300 mM imidazole, 10% glycerol). Fractions containing the protein of interest were combined and dialyzed into 20 mM HEPES pH 7.3, 150 mM NaCl, and 10% glycerol.

### Thermal shift assays

To determine the melting temperature (T_m_) of the CARF domains of *Bb*Chp and *Ps*Chp, a thermal shift assay was performed using kit 4462263 from Thermo Fisher. 5 μM of purified *Bb*Chp (307–528) or *Ps*Chp (295–523) was co-incubated with either 100 µM of cA_3_, cA_4_, cA_6_, or no ligand. Fluorescence was measured using the Melt Curve program on the QuantStudio 3 Real-Time PCR System (Applied Biosystems) as the mixture was heated at a rate of 0.1°C/s from 25°C to 98°C. The first derivative of the relative fluorescence units (RFU) was plotted over temperature to determine T_m_ for each condition. To determine the dissociation constant (K_d_), 5 µM of purified *Bb*Chp (307–528) was co-incubated with two-fold dilutions of cA_4_ (from 100 µM to 0.76 nM) and heated as described earlier. The first derivatives of the RFU at 41°C, the apo T_m_, were normalized against each other from 0 to 1 using the equation: (RFU – min RFU)/(max RFU – min RFU). These values were then plotted against cA_4_ concentration and fit using a nonlinear regression for one-site binding to determine the K_d_ value.

### Ring nuclease assay

To monitor ring nuclease activity of the CARF domain of *Bb*Chp or *Ps*Chp, 500 mM of cA_4_ alone or with either 2 mM of *Bb*Chp CARF domain or *Ps*Chp domain was incubated in reaction buffer (20 mM HEPES pH 7.1, 150 mM NaCl, 1 mM TCEP, 1 mM MnCl_2_ and 10% glycerol). Reactions were carried out at 37° for 18 h before filtering the reaction products and collecting chromatograms on the HPLC using the same method described previously.

### Analysis of common nucleotides and nucleosides using HPLC-coupled high-resolution mass spectrometry

Colonies containing pTarget and pCRISPR plasmids were launched overnight. The following day, cultures were diluted 1:100, grown to OD_600_ of 0.3, and induced with 125 ng/mL aTc for 15 min before pelleting and collection. Cells were resuspended in 4 mM ethylenediaminetetraacetic acid (EDTA) dissolved in 80% ethanol and vortexed vigorously. Next, cells were heated to 85°C with shaking for 3 min and allowed to cool to room temperature. Cell lysates were spun down at 13 000 rpm in a tabletop centrifuge and supernatants were then further processed. About 600 μL of supernatant was dried under blowing air at room temperature for 2 h. The remaining extracts were reconstituted to 100 μL with 10 mM ammonium bicarbonate solution (pH 7) and run through 10 kDa filters at 14 000 × *g*, 4°C for 30 min. The filtrates and chemical standards of common nucleotides and nucleotides were analyzed on Sciex X500 QTOF mass spectrometer with electrospray ionization in the negative mode. Samples were delivered by ExionLC AC system equipped with ACQUITY UPLC HSS T3 column (2.1 × 100 mm, 1.8 μm) that was held at 40°C. Solvent A was water with 10 mM ammonium acetate (pH 6.9) and solvent B was acetonitrile. Flow rate was 0.5 mL/min. For each run, 1 μL of sample was injected and separated with the following gradient: 0% B for 1.5 min, to 30.0% B in 3.5 min, to 95% B in 1.0 min, and 95% B for 1.0 min. Column eluate between 0.5 min and 5 min was directed to MS using the following method: curtain gas of 30 psi, ion source gas 1 of 50 psi, ion source gas 2 of 50 psi, temperature of 400°C, and soray voltage of 550. For TOFMS, m/z between 100 and 1000 was scanned with a declustering potential of 80 V, collision energy of 10 V, collision gas of 7 psi, and accumulation time of 0.1 s. For TOFMSMS, MRM (Multiple Reaction Monitoring) was applied: declustering potential of 80 V, collision energy of 35 V, and accumulation time of 0.03 s. All data were collected and analyzed on the software Sciex OS. The levels of common nucleotides and nucleosides were semi-quantified using the peak intensity of each molecule’s characteristic MRM transition.

### *In vitro* dephosphorylation reactions

Dephosphorylation reactions were performed *in vitro* by incubating substrates at 37°C for 90 min. Reaction substrates were incubated at a final concentration of 1 mM with 50 µM *Bb*Chp (1–168) or *Ps*Chp (1–138) HAD domains in reaction buffer [20 mM HEPES pH 7.1, 150 mM NaCl, 1 mM TCEP, 1 mM of metal (MnCl_2_, MgCl_2_, CoCl_2_, NiSO_4_, CaCl_2_, CuSO_4_, and 10% glycerol]. After incubation at 37°C, reactions were quenched by heating mixtures to 65°C for 5 min. Reaction mixtures were diluted with nuclease-free water to 100 μL before being filtered with Amicon^®^ Ultra Centrifugal Filter, 10 kDa MWCO filters to remove proteins before analysis. 10 μL of filtered reaction products were then injected onto an Agilent Bonus-RP, 4.6 × 150 mm, 3.5 μm Rapid Res. C18 column held at 40°C at a flow rate of 1.2 mL per min. For ATP, ADP, AMP, IMP, inosine, dATP, and dADP, the following mobile phase buffer was used in the elution program: 60 mM K_2_HPO_4_, 40 mM KH_2_PO_4_, pH 7.0. For GTP, GDP, CTP, CDP, UTP, and UDP, 10 mM TBAB was supplemented into the mobile phase buffer. The elution program was as follows: 0 min 100% buffer, 0% ACN; 2 min 95% buffer, 5% ACN; 4 min 80% buffer, 20% ACN; 5.3 min 75% buffer, 25% ACN; and 6 min 100% buffer, 0% ACN. Chromatograms were collected by monitoring absorbance at 254 nm. To determine substrate conversion, integrated peaks from reaction mixtures without EDTA were compared to integrated peaks from reaction mixtures with EDTA.

### Free phosphate assay

Free phosphate reactions were performed *in vitro* by incubating substrates at 37°C for 90 min. Reaction substrates were incubated at a final concentration of 1 mM with 50 µM *Bb*Chp (1–168) or *Ps*Chp (1–138) HAD domains in reaction buffer (20 mM HEPES pH 7.1, 150 mM NaCl, 1 mM TCEP, 1 mM MnCl_2_, and 10% glycerol). After incubation at 37°C, reactions were quenched by heating mixtures to 65°C for 5 min. A mixture of 30 μL of reaction mixture, 20 μL of sterile water, and 100 μL Malachite Green (Phosphate Assay Kit, Sigma MAK308) was incubated at room temperature for 30 min and then imaged at OD_620_ using a plate reader. A standard curve was generated using phosphate standards (Phosphate Assay Kit, Sigma MAK308) and used to calculate the concentration of free phosphate for experimental conditions.

### Structural predictions

Structural predictions were done using AlphaFold3 [38]. The obtained models are available in ModelArchive (modelarchive.org) with the accession codes ma-dddsx, ma-qx52m, ma-2js6x, and ma-8h8m9.

## Results

### *Bb*Chp reduces ATP and IMP levels to mediate cell toxicity during the type III-A CRISPR–Cas response

**Figure 1.**
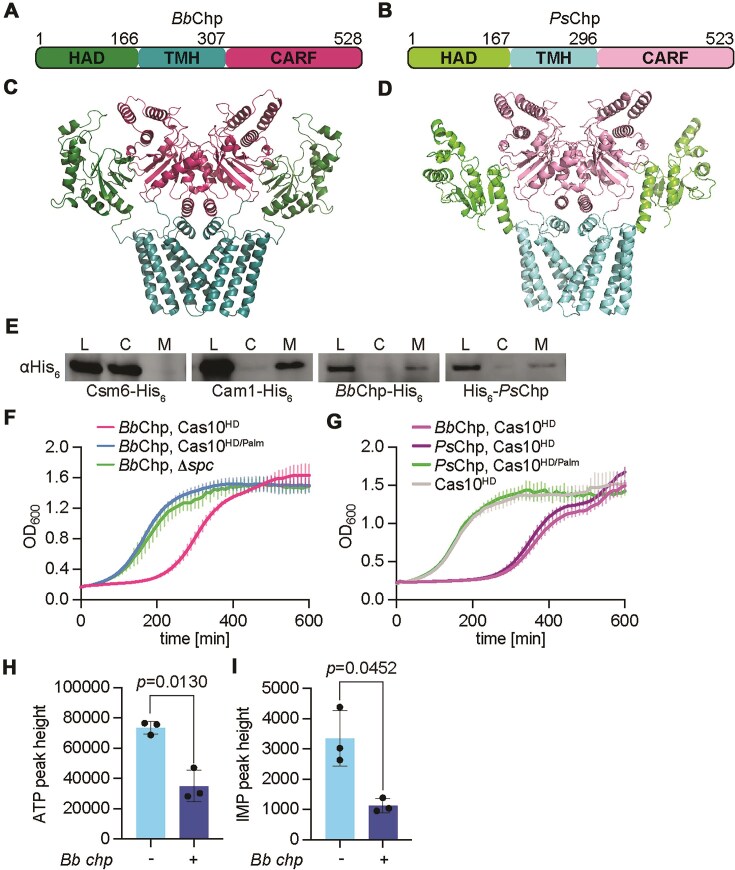
Activation of Chp effectors leads to cell toxicity and reduced ATP and IMP levels *in vivo*. (**A**) Domain architecture of *Bacteroidales* bacteria Chp (*Bb*Chp), which was identified within a type III-D CRISPR–Cas locus. (**B**) Domain architecture of *Prevotella sp*. Chp (*Ps*Chp), which was identified within a type III-A CRISPR–Cas locus. (**C**) AlphaFold3 structure of *Bb*Chp. The protein contains an N-terminal HAD phosphatase domain (green) followed by transmembrane helices (blue) and a C-terminal CARF domain (pink). (**D**) AlphaFold3 structure of *Ps*Chp. The protein contains an N-terminal HAD phosphatase domain (light green) followed by transmembrane helices (light blue) and a C-terminal CARF domain (light pink). Residue numbers are labeled. (**E**) Western blot of cellular fractions. Staphylococci harboring plasmids encoding hexahistidine-tagged Csm6, Cam1, *Bb*Chp, and *Ps*Chp were outgrown and lysed. Total lysate fractions (L) were collected and then subjected to ultracentrifugation to obtain cytosolic (C) and membrane (M) fractions. Samples were blotted with a primary anti-His_6_ antibody. (**F, G**) Growth of staphylococci carrying pTarget and pCRISPR variants, measured as OD_600_ after the addition of aTc. Data are mean of three biological triplicates ± SEM. (**H**) Quantification of ATP and (**I**) IMP levels from bacterial lysates. Extracts from staphylococci harboring pTarget and pCRISPR(ΔChp) or pCRISPR(*Bb*Chp) were collected after 15 min of incubation with aTc and analyzed via liquid chromatography–mass spectrometry (LC–MS). Mean of three biological replicates ± SEM is reported. The *P-*values shown were obtained with a two-sided *t*-test with Welch’s correction.

A previous bioinformatic search for novel CRISPR-associated genes [23] led to the identification of two genes encoding an N-terminal HAD phosphatase domain followed by four predicted transmembrane helices and a C-terminal CARF domain. We named these genes CRISPR-associated HAD phosphatases (Chp). One of them is located within a type III-D CRISPR–Cas locus from an unidentified *Bacteroidales* bacterium (*Bb*Chp, Fig. 1A and Supplementary Fig. S1A) and the other exists within a type III-A locus present in *Prevotella sp*. (*Ps*Chp, Fig. 1B and Supplementary Fig. S1A). A structural prediction of *Bb*Chp made using AlphaFold3 [38] suggests the formation of a dimer through the interaction between the CARF domains of two *Bb*Chp monomers (Fig. 1C). In support of this prediction, most CARF effectors whose structures have been determined to date have a dimeric architecture to generate a symmetric binding site for cA_4_ or cA_6_ [4]. Additional contacts between the α-helices of two different subunits seem to reinforce the dimeric structure. In contrast, HAD phosphatase domains are not predicted to interact within the dimer. *Ps*Chp shares 84% sequence similarity with *Bb*Chp (Supplementary Fig. S1B) and is predicted to have an equivalent structure (Fig. 1D).

To investigate the role of these CARF effectors in the type III CRISPR–Cas response, we cloned each of them individually into pCRISPR, a plasmid harboring the *Staphylococcus epidermidis* RP62a type III-A CRISPR–Cas locus [39], which is genetically related to the type III systems that naturally harbor both *chp* genes (Supplementary Fig. S1B). In this plasmid we replaced the open reading frame encoding for the staphylococcal CARF effector Csm6 with either of the *chp* genes, generating pCRISPR(Chp) (Supplementary Fig. S1C). Using this construct to express *Bb*Chp and *Ps*Chp in staphylococci, we tested their subcellular localization through the introduction of a C- and N-terminal hexa-histidyl tag, respectively. Expression of C-terminally tagged Csm6 and Cam1 served as cytoplasmic and membrane-bound controls, respectively [12]. We obtained staphylococcal lysates expressing these different CARF effectors and performed an anti-hexa-histidine western blot (Fig. 1E and Supplementary Fig. S1D) and found that both *Bb*Chp and *Ps*Chp primarily associate with the cell membrane, most likely via the predicted transmembrane helices.

We previously investigated the function of CARF effectors through the evaluation of cell toxicity and growth arrest induced upon activation of the Cas10 complex by a target RNA [11–14, 40]. We therefore generated an *S. aureus* RN4220 strain [41] harboring both pCRISPR(*Bb*Chp) and pTarget [14]. pTarget encodes a target RNA under a tetracycline-inducible promoter. The Cas10 complex is expressed from pCRISPR(*Bb*Chp) and programmed with a crRNA complementary to target RNA. To prevent pTarget degradation by the nuclease activity of the Cas10 complex [14], we introduced mutations in the HD domain (H14A, D15A) of the Cas10 subunit, generating pCRISPR(*Bb*Chp, Cas10^HD^). To test for *Bb*Chp toxicity, we treated cultures with anhydrotetracycline (aTc) to induce target RNA expression and production of cOAs by the Cas10 complex and followed bacterial growth by measuring optical density at 600 nm (OD_600_). We found that, compared to control cultures that do not produce cOAs due to the absence of either a crRNA that can recognize the target (*Bb*Chp, Δ*spc*) or cOA synthesis (mutations in the cyclase active site of Cas10; D586A, D587A; Cas10^Palm^), the growth of staphylococci carrying *Bb*Chp was delayed (Fig. 1F). This growth arrest depended on the presence of the CARF effector, since it was not detected in cells harboring a plasmid that lacks a CARF effector [pCRISPR(Cas10^HD^)] (Fig. 1G). A test of *Ps*Chp in similar genetic backgrounds showed that this CARF-HAD phosphatase variant also generates a growth arrest upon production of cOAs during the staphylococcal type III-A CRISPR–Cas response (Fig. 1G).

Given that some of the most common substrates of HAD phosphatases are NTPs [30], and that a common anti-phage defense strategy is the alteration of the nucleotide pool of the host to prevent viral propagation [42–45], we decided to investigate the effect of *Bb*Chp activation on the abundance of different nucleotides within staphylococci, using untargeted LC/MS analysis. We collected cells from cultures either expressing [pCRISPR(Cas10^HD^, *Bb*Chp)] or lacking [pCRISPR(Cas10^HD^, ΔChp)] *Bb*Chp, in which cOA production was induced by the addition of aTc for 15 min, and quantified 27 different nucleotides in each sample. We observed that *Bb*Chp activation resulted in a significant decrease in ATP (Fig. 1H) and IMP levels (Fig. 1I) but not for other nucleotides (Supplementary Fig. S2). Together this data demonstrates that *Bb*Chp activation during the type III-A CRISPR–Cas response leads to a reduction of ATP and IMP levels and causes a growth arrest in staphylococci. We hypothesized that this is a consequence of (i) the binding of cOA second messengers to the CARF domain of the Chp effectors that (ii) stimulate the nucleotide phosphatase activity of the HAD domain. We therefore performed biochemical experiments to test these hypotheses.

### The CARF domains of *Bb*Chp and *Ps*Chp bind cA_4_

**Figure 2.**
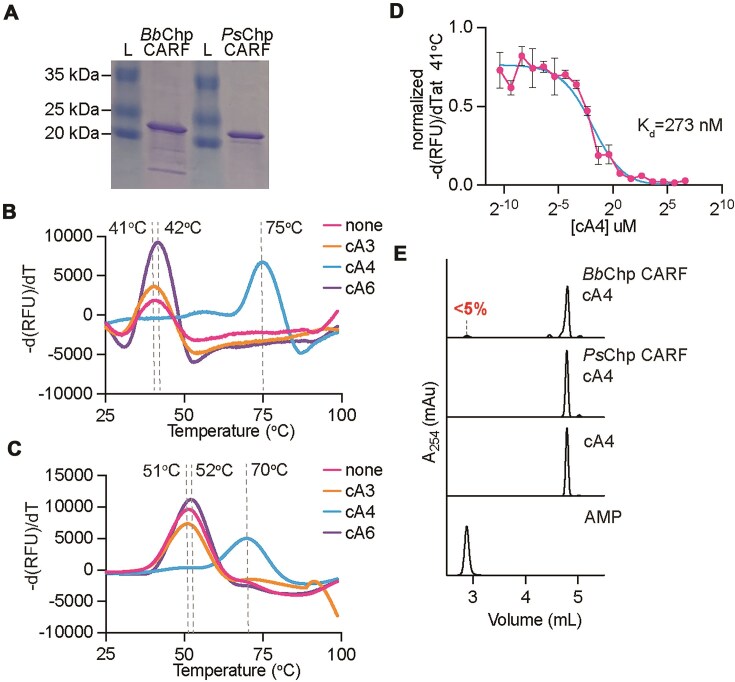
The *Bb*Chp and *Ps*Chp CARF domains bind cA_4_. (**A**) Sodium dodecyl sulfate–polyacrylamide gel electrophoresis (SDS–PAGE) stained with Coomassie showing purified *Bb*Chp CARF domain and purified *Ps*Chp CARF domain. (**B**) Thermal shift assay of purified *Bb*Chp CARF domain (5 µM) incubated with cAn ligand (100 µM) or no ligand. First derivative of RFU plotted as a function of temperature. Mean of three technical triplicates is reported. (**C**) Same as (B) but with purified *Ps*Chp CARF domain. Mean of two technical duplicates is reported. (**D**) Thermal shift assay of purified *Bb*Chp CARF domain (5 µM) incubated with varying concentrations of cA_4_. The first derivative of RFU at 41°C was normalized and then plotted as a function of cA_4_ concentration. A nonlinear regression for one-site binding (saturation) was performed to calculate K_d_. Mean of three technical triplicates, ± SEM, is reported. (**E**) HPLC analysis of ring nuclease reaction products of CARF domain (2 mM) incubated with cA_4_ (500 mM). Chromatograms of cA_4_ and AMP are shown as standards. Reactions were performed in duplicate.

To determine whether *Bb*Chp and *Ps*Chp bind any of the cOAs produced by the cyclase activity of the Cas10 RNA-guided complex, cA_3_, cA_4_, and cA_6_ [7, 8], we decided to purify both proteins using heterologous expression in *E. coli*. However, most likely due to the presence of the transmembrane helices, we found the proteins to be insoluble. We therefore expressed and purified the N-terminal hexa-histidyl tagged CARF domains of both Chp effectors (Fig. 2A). We then performed thermal shift assays, mixing the purified CARF domains (5 mM) with 100 mM of either cA_3_, cA_4_, or cA_6_ and a fluorescent dye that binds to exposed hydrophobic residues to monitor protein thermal stability, and followed the change in fluorescence during heating of the mixture. We calculated the first derivative of RFU [−d(RFU)/dT] to determine the protein melting temperature values (T_m_) in the presence of each cOA (Fig. 2B). We found that the T_m_ of *Bb*Chp–CARF was 41°C either in the absence of ligand or in the presence of cA_3_, and 42°C in the presence of cA_6_, indicating these cOAs do not stabilize the protein. In contrast, when *Bb*Chp–CARF was incubated with cA_4_ the T_m_ shifted to 75°C, an observation that demonstrates that tetra-adenylate is the ligand of *Bb*Chp. Similar results were obtained with purified *Ps*Chp-CARF (Fig. 2A), with a T_m_ of 51°C either in the absence of ligand or in the presence of cA_3_, 52°C in the presence of cA_6_, but 70°C when incubated with cA_4_ (Fig. 2C). We also calculated the binding affinity of *Bb*Chp–CARF for its ligand by incubating 5 mM of the protein with varying concentrations of cA_4_ and fluorescent dye and monitoring the change in fluorescence upon heating. We then normalized the −d(RFU)/dT measurements obtained at 41°C (the T_m_ of apo *Bb*Chp–CARF) for the different ligand concentrations (Fig. 2D). As a consequence of the shift of the T_m_ to 75°C upon ligand binding, the normalized −d(RFU)/dT values decreased as the cA_4_ concentration increased. We performed a nonlinear regression to fit a binding curve to our data, which was used to determine the equilibrium dissociation constant (K_d_) of 273 nM for the binding of cA_4_ to *Bb*Chp–CARF. These data demonstrate that the CARF domains of both *Bb*Chp and *Ps*Chp bind cyclic tetra-adenylates. Finally, to determine whether *Bb*Chp or *Ps*Chp CARF domains display ring nuclease activity, which was found in several other CARF effectors [46, 47], we incubated the purified CARF domains with cA_4_ overnight and separated the reaction products using HPLC. We found that neither *Bb*Chp CARF nor *Ps*Chp CARF displays significant ring nuclease activity (Fig. 2E).

### Chp effectors dephosphorylate ATP and dATP

**Figure 3.**
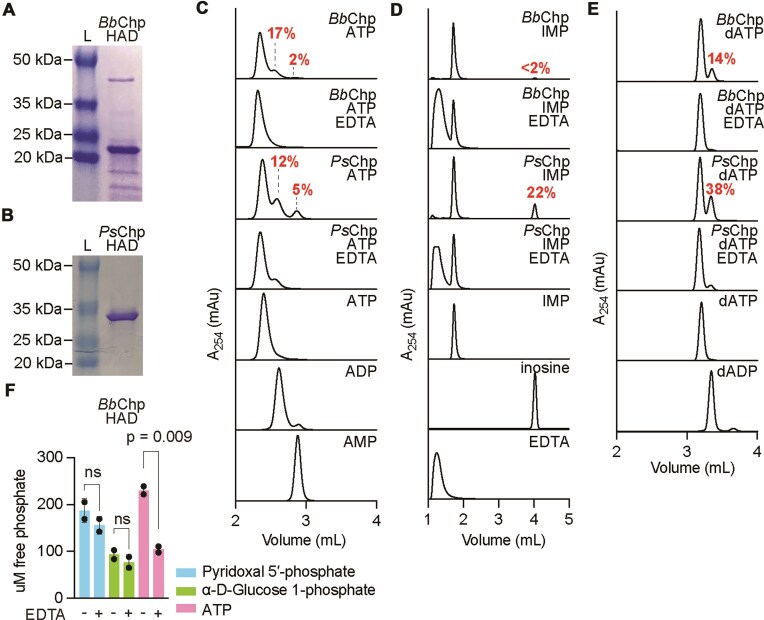
Chp HAD domains dephosphorylate nucleotides. (**A, B**) SDS–PAGE stained with Coomassie Blue showing purified *Bb*Chp and *Ps*Chp HAD domains. (**C**) HPLC analysis of *Bb*Chp (50 mM) or PsChp (50 mM) reaction products in the presence of ATP (1 mM). Chromatograms of ATP, ADP, and AMP are shown as standards. Reactions were performed in duplicate. Percentage of substrate conversion was calculated by comparing peak integration values of reactions lacking EDTA to reactions containing EDTA. Average percentage from two duplicates is shown. (**D**) Same as with (C) but in the presence of IMP (1 mM). Chromatograms of IMP, inosine, and EDTA are shown as standards. (**E**) Same as with (C) but in the presence of dATP (1 mM). Chromatograms of dATP and dADP are shown as standards. (**F**) Free phosphate assay for *Bb*Chp HAD (50 mM) in the presence of pyridoxal 5′-phosphate, α-d-glucose 1-phosphate, or ATP (1 mM). The mean of two biological replicates, ± SD, is reported. The *P-*values shown were obtained with a two-sided *t-*test with Welch’s correction.

Given the reduction in ATP and IMP levels that we detected *in vivo* upon activation of *Bb*Chp and *Ps*Chp (Fig. 1H and I), and that nucleotides are common substrates of HAD phosphatases [30], we decided to test for the enzymatic activity of *Bb*Chp and *Ps*Chp on a diverse set of nucleotides. To do this, we purified an N-terminal hexa-histidyl tagged version of the HAD phosphatase domain of *Ps*Chp and *Bb*Chp (Fig. 3A and B). We incubated *Ps*Chp-HAD or *Bb*Chp-HAD (50 mM) with ATP, IMP, UTP, CTP, GTP, TTP, dCTP, dGTP, or dATP (1 mM), Mn^2+^ (1 mM), in the presence or absence of EDTA, and separated the reaction products using HPLC. We were unable to detect changes for UTP, CTP, GTP, TTP, dCTP, or dGTP (Supplementary Fig. S3). In contrast, for *Ps*Chp-HAD we observed conversion of dATP to dADP (38%), ATP to ADP and AMP (12% and 5%, respectively), and IMP to inosine (22%) (Fig. 3C–E). For *Bb*Chp-HAD we observed conversion of dATP to dADP (14%), ATP to ADP and AMP (17% and 2%, respectively), but only minimal phosphatase activity on IMP (<2% inosine) (Fig. 3C–E). EDTA prevented this conversion, a result that indicates that Mn^2+^ is required for the reaction, similar to other HAD phosphatases [30, 48]. We also tested the activity in the presence of other divalent cations. We found that *Ps*Chp-HAD is able to dephosphorylate dATP using Mg^2+^ (40%) and Co^2+^ (33%) but displayed less activity in the presence of Ni^2+^ (22%), Ca^2+^ (12%), and Cu^2+^ (6%) (Supplementary Fig. S4A). Given the high levels of dephosphorylation observed with Mg^2+^ on dATP, we tested a series of different nucleotides and found activity on ATP (Supplementary Fig. S4B; 16% ADP, 5% AMP) but not on IMP (Supplementary Fig. S4C), as was the case in the presence of Mn^2+^, a result that indicates that the divalent cation can affect substrate specificity. We did not detect dephosphorylation of UTP, CTP, GTP, TTP, dCTP, or dGTP (Supplementary Fig. S4D–I). Finally, we tested whether the *Bb*Chp-HAD domain can dephosphorylate non-nucleotide substrates using a colorimetric assay that measures the release of free phosphate groups. We incubated *Bb*Chp-HAD with either ATP (as a positive control), pyridoxal 5′-phosphate (a flavin derivative), or α–d–glucose 1-phosphate. No free phosphate was detected for these alternative substrates (Fig. 3F), indicating that the *Bb*Chp-HAD dephosphorylation may be specific to nucleotides.

These experiments demonstrate that *Bb*Chp and *Ps*Chp have phosphatase activity *in vitro*. In addition, degradation of ATP and IMP for *Ps*Chp correlates with the *in vivo* LC/MS results obtained after activation of *Bb*Chp *in vivo*, for which we observed a decrease in the cellular levels of these two nucleotides (Fig. 1H and I). While *Bb*Chp does not seem to dephosphorylate IMP *in vitro*, we confirmed it specifically dephosphorylates nucleotides rather than working as a general HAD phosphatase.

### Conserved residues found in HAD phosphatases are not essential for *Bb*Chp toxicity

**Figure 4.**
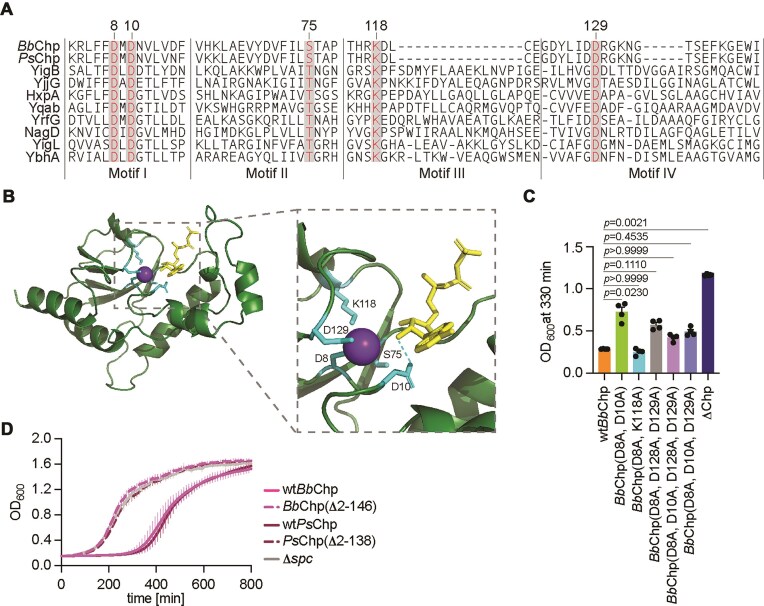
Mutational analysis of conserved residues in *Bb*Chp HAD domain. (**A**) Protein alignment of known motifs from *E. coli* HAD phosphatases with *Bb*Chp and *Ps*Chp. Highly conserved residues are highlighted. Amino acid positions of *Bb*Chp residues are shown at the top. (**B**) AlphaFold3 structure of HAD phosphatase domain from *Bb*Chp with conserved residues noted. Modeled with ATP and Mn^2+^. (**C**) Growth of staphylococci harboring pTarget and pCRISPR(*Bb*Chp) with mutations in highly conserved HAD residues measured as OD_600_ value at 330 min after addition of aTc. Data are mean of two biological duplicates ± SEM. The *P*-values shown were obtained with an ANOVA test. (**D**) Growth of staphylococci carrying pTarget and pCRISPR variants, including deletion of HAD domains in *Bb*Chp and *Ps*Chp, measured as OD_600_ after the addition of aTc. Data are mean of two biological triplicates ± SEM.

A previous bioinformatic analysis of HAD hydrolases indicated that although they share little overall sequence similarity (15%–30%), they contain four conserved motifs, I–IV [25, 49, 50]. Motif I contains a signature DxD sequence, in which the aspartic acids coordinate the metal cofactor required for hydrolysis. The first aspartate acts as a nucleophile and forms an aspartyl intermediate during catalysis [51, 52]. In the case of HAD phosphatases, the second aspartate acts as a general acid-base. Motifs II and III contain highly conserved threonine or serine and lysine, respectively, and may contribute to the stability of the reaction intermediates during hydrolysis [49]. Motif IV contains acidic residues with the signatures DD, GDxxxD, or GDxxxxD, where the underlined aspartate is highly conserved and also contributes to the coordination of the metal cofactor [52, 53]. We aligned *Bb*Chp and *Ps*Chp to 10 HAD hydrolase sequences that were previously characterized bioinformatically using MUSCLE [30, 36], and found that they contain motifs I, II, and IV, as well as a minimal motif III (Fig. 4A). Moreover, *Bb*Chp contains all five highly conserved residues: D8, D10, S75, K118, and D129. A structural prediction of the HAD phosphatase domain of *Bb*Chp in the presence of ATP made using AlphaFold3 [38] suggests that these residues surround a metal ion (Fig. 4B). To test the importance of these residues for *Bb*Chp activity, we introduced alanine substitutions to generate the following mutants: D8A/D10A, D8A/K118A, D8A/D10A/D129A, D8A/D128A/D129A, and D8A/D10A/D128A/D129A. We then tested *Bb*Chp’s ability to induce a growth arrest in staphylococci during the activation of type III-A CRISPR–Cas immunity (Supplementary Figs S5A and S4C). Surprisingly, we found that none of the substitutions abolished the growth delay caused by wild-type *Bb*Chp; only mutations D8A/D10A and D8A/D128A/D129A displayed a partial reduction in toxicity (Fig. 4C). We also tested the effect of these amino acid substitutions *in vitro*, after the purification of mutant *Bb*Chp HAD domains (Supplementary Fig. S5B). In all cases we observed reduced phosphatase activity on ATP (from 17% to 6%–0%) and dATP (from 14% to 8%–4%), with exception of the D8A/K118A mutant, which displayed a minimal reduction (Supplementary Fig. S5C–G). These results suggest that even weak dephosphorylation activity may be sufficient to induce toxicity within staphylococcal cells. Finally, we made deletions that encompass the entire HAD phosphatase domain for both CARF effectors, generating *Bb*Chp(Δ2–146) and *Ps*Chp(Δ2–138), which in both cases eliminated Chp toxicity (Fig. 4D).

### *Bb*Chp and *Ps*Chp provide anti-phage defense

**Figure 5.**
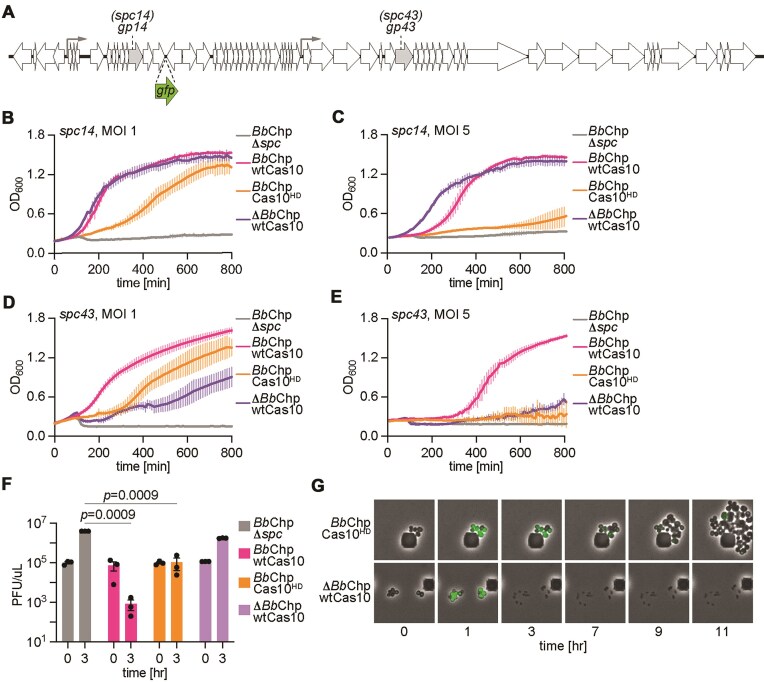
*Bb*Chp and *Ps*Chp are required for anti-phage defense. (**A**) Schematic of the genome of staphylococcal phage ϕNM1γ6 showing the locations of the transcripts targeted by different spacers of the type III-A CRISPR–Cas system. Gray arrows indicate promoters. GFP is inserted at indicated location for fluorescent microscopy experiments. (**B**) Growth of staphylococci carrying different pCRISPR constructs with a spacer to target the *gp14* transcript of ϕNM1γ6. Measured OD_600_ with ϕNM1γ6 at an MOI of 1. Mean of three biological triplicates, ± SEM, is reported. (**C**) same as in panel (B) at an MOI of 5. (**D**) same as in panel (B) but targeting the *gp43* transcript. (**E**) same as in panel (D) but at an MOI of 5. (**F**) Enumeration of PFU within staphylococcal cultures harboring different pCRISPR constructs with a spacer to target the *gp43* transcript at the indicated times after infection with ϕNM1γ6 at an MOI of 1. The mean of three biological replicates, ± SEM, is reported. The *P-*values shown were obtained with a two-sided *t*-test with Welch’s correction. (**G**) Time course microscopy of *S. aureus* encoding different pCRISPR constructs after infection with ϕNM1γ6-GFP. Images are representative of two biological replicates.

For the majority of CARF effectors studied to date, the toxicity they mediate is essential to provide anti-phage immunity when the target transcript is expressed late during the viral lytic cycle and Cas10’s nuclease activity is not sufficient to restrict infection [9–13, 40]. We tested the function of *Bb*Chp in the type III-A CRISPR–Cas response after infection with the staphylococcal phage ϕNM1γ6 at multiplicity of infection (MOI) 1 and 5. We programmed the Cas10 complex with crRNA guides that recognize either the early or late transcripts of the *gp14* or *gp43* genes, respectively (the crRNAs originated from *spc14* and *spc43*, respectively) (Fig. 5A). As reported before for other CARF effectors [9, 11–13, 40], immunity mediated by *spc14* did not require *Bb*Chp but depended on the nuclease activity of Cas10, both at MOI 1 (Fig. 5B) and 5 (Fig. 5C). In the absence of Cas10 nuclease activity, however, *Bb*Chp alone was able to support immunity at MOI 1 (Fig. 5B) but not at 5 (Fig. 5C), consistent with an abortive infection mode of defense. Interestingly, the activation of *Bb*Chp early during infection generated a noticeable growth delay at MOI 1, which provides further evidence that *Bb*Chp induces growth arrest during phage infection. In contrast, *Bb*Chp was sufficient to provide defense mediated by *spc43* at MOI 1, with staphylococci proliferating with a marked delay (Fig. 5D). At MOI 5, *Bb*Chp alone did not enable the survival of the bacterial culture, and required the nuclease activity of Cas10 (Fig. 5E), a similar result to that obtained with other CARF effectors heterologously expressed in staphylococci such as Cam1 [12] and Cad1 [13]. These results were corroborated by enumerating PFU 3 h after infection with ϕNM1γ6 at MOI 1 (Fig. 5F). We found that, when type III-A CRISPR immunity is triggered late in the phage lytic cycle via *spc43, Bb*Chp, but not Cas10, alone prevented the increase in PFU values observed in the absence of CRISPR immunity (Δ*spc*). In contrast, the presence of both *Bb*Chp and the nuclease activity of Cas10 caused a marked decrease of viral propagation. To investigate these results at the cellular level, we performed fluorescence microscopy, taking advantage of GFP expression by ϕNM1γ6^GFP^ to visualize infected cells [9]. Consistent with the growth arrest mediated by *Bb*Chp activation that we observed in the presence of both pTarget (Fig. 1F) and phage (Fig. 5D), infection of cultures carrying a type III-A CRISPR–Cas system programmed *spc43* and expressing *Bb*Chp and Cas10^HD^ led to a stop in the division of the infected cells (marked by green fluorescence) that was followed by the division of non-infected ones (non-fluorescent) (Fig. 5G). In contrast, in the absence of *Bb*Chp, the green infected cells lysed. Based on these data, we conclude that the growth arrest generated by *Bb*Chp affects the ability of ϕNM1γ6 to complete its infectious cycle and the release of viral particles from infected hosts, allowing non-infected cells to proliferate. Finally, we investigated the importance of conserved HAD domain residues for the defense provided by both *Bb*Chp and *Ps*Chp. *Ps*Chp also contains the same conserved residues (Fig. 4A) forming a similarly predicted metal ion pocket as *Bb*Chp (Supplementary Fig. S6A). We tested the D8A/D10A, D8A/K118A, D8A/D10A/D129A, D8A/D128A/D129A, and D8A/D10A/D128A/D129A substitutions in *Bb*Chp and D9A/D11A, D9A/K112A, and D9A/D11A/D123A in *Ps*Chp. Both by measuring the growth of infected cultures (Supplementary Fig. S6B and C) as well as plaque formation (Supplementary Fig. S6D and E), we found that these mutations had minimal, if any, effect on immunity. Only inactivation of the cyclase active site of Cas10 (dPalm) or the complete deletion of the HAD domains abrogated immunity. Given the reduction of phosphatase activity detected after mutation of *Bb*Chp HAD domain (Supplementary Fig. S5C–G), these results suggest that reduced dephosphorylation activity may be sufficient to disrupt phage replication.

## Discussion

Here we investigated the role of CARF effectors harboring HAD phosphatase domains in the staphylococcal type III-A CRISPR–Cas immune response. We found that two highly similar homologs, *Bb*Chp and *Ps*Chp, localize to the bacterial membrane, and upon activation of the Cas10 complex by a target transcript, bind cA_4_ cyclic nucleotides and cause HAD phosphatase-dependent cytotoxicity to prevent viral propagation. The impossibility of purifying full-length *Bb*Chp or *Ps*Chp, most likely due to the presence of the transmembrane helices, prevented us from demonstrating a direct stimulation of the phosphatase activity after cA_4_ binding. However, we have two reasons to believe this is likely. First, although we were not able to test cyclic oligoadenylate binding by the full-length proteins, previous studies have shown an excellent correlation of the ligand specificity and binding between purified CARF domains and full-length effectors [13]. Second, we determined that cell toxicity is triggered by cA_4_ synthesis *in vivo* and depends on the presence of the HAD phosphatase domain of *Bb*Chp and *Ps*Chp. Based on these observations, we propose that ligand binding by the CARF domain results in a conformational change that stimulates the HAD phosphatase activity of *Bb*Chp and *Ps*Chp.

How these CARF effectors cause toxicity and provide immunity is less clear. Several bacterial anti-phage defense systems deplete nucleotides to halt the lytic cycle and prevent viral propagation. For example, cytidine-deaminases deplete dCTP by converting it to dUTP [43, 45]; effectors associated with cyclic-oligonucleotide-based anti-phage signaling systems (CBASS) possess ATP nucleosidase activity that cleaves this fundamental nucleotide into adenine and ribose-5′-triphosphate [44]; and GajB, the effector of the Gabija system, has phosphatase activity that hydrolyzes and depletes ATP, GTP, dATP, and dGTP [42]. Given these precedents, that some of the most common substrates of HAD phosphatases are NTPs [30], and that our *in vitro* and *in vivo* data demonstrated NTP dephosphorylation and nucleotide depletion, respectively, we hypothesized that *Bb*Chp and *Ps*Chp provide defense through the hydrolysis of phosphate groups from nucleotides, leading to a decrease in the cellular levels of these important metabolites. More specifically, we observed dephosphorylation of ATP, dATP, and IMP *in vitro* and detected depletion of ATP and IMP, but not dATP, *in vivo*. dATP is generated directly and indirectly from ATP molecules by ATP reduction by ribonucleotide reductases [54] or phosphorylation of dAMP and dADP using ATP as the donor of phosphate groups by nucleoside kinases [55]. Therefore, we believe that it is possible that *Bb*Chp and *Ps*Chp also dephosphorylate dATP *in vivo*, but that the reduction of dATP is compensated by consuming ATP molecules, a scenario consistent with our results. Importantly, we explored the activity of *Bb*Chp-HAD on other common HAD phosphatase substrates such as pyridoxal 5′-phosphate (PLP) and phosphorylated carbohydrates [30]. In principle, degradation of these or other phosphorylated metabolites could cause the reduction in cell growth and phage propagation we detected. Therefore, in spite of the seemingly specific nature of *Bb*Chp for nucleotide dephosphorylation, it is possible that phosphorylated substrates other than nucleotides are additional targets of *Bb*Chp and *Ps*Chp during the type III-A CRISPR–Cas response. This is a necessary consideration given that our *in vitro* assays were performed with only the HAD phosphatase domains of the Chp effectors, outside of the membrane localization context and cA_4_-mediated activation.

The biological importance of the transmembrane helices of *Bb*Chp and *Ps*Chp remains to be understood. Most surface-located 5′-nucleotidases hydrolyze AMP, ADP, and ATP and are employed by pathogenic bacteria to increase the extracellular concentrations of adenosine, an immunosuppressive molecule that helps evade the host’s immune response [56, 57]. In contrast, intracellular nucleotidases can regulate the nucleotide pool for DNA and RNA synthesis [57, 58]. Given that activation of *Bb*Chp and *Ps*Chp depends on the production of intracellular cOA, it is reasonable to assume that both the CARF and HAD domains are located inside the host cell and therefore alter intracellular nucleotide levels upon induction. We believe that the transmembrane helices may be a key structural feature that allows the CARF domains to dimerize and form protein–protein contacts with the HAD domains for allosteric regulation. We acknowledge that an opposite scenario is also possible: HAD domains may be important to transduce a conformational change to the transmembrane helices upon ligand binding that triggers membrane disruption. In this case the dephosphorylation activity of Chp effectors would not cause cell toxicity, a hypothesis that is in line with our finding that deletion of the full HAD domain, but not amino acid substitutions, abrogates growth arrest and anti-phage defense.

Whether by depleting nucleotides or other phosphorylated metabolites, *Bb*Chp and *Ps*Chp activation during the type III-A response generates a marked growth arrest that is important to provide immunity against phage infection. Like other CARF effectors, this mode of defense is most important when the target transcript is expressed late in the viral lytic cycle and in the absence of Cas10 ssDNase activity. This is especially important because approximately only 40% of type III CRISPR–Cas systems contain variants that either lack an HD domain [59] or do not display detectable ssDNase activity [60]. When compared with other CARF effectors, *Bb*Chp and *Ps*Chp are unique in that they cause ATP depletion, and therefore it is intriguing to think that their activity may decrease cyclic oligoadenylate synthesis, which requires ATP [61], and negatively impact the immune response they mediate. CARF effectors display a great diversity of mechanisms to halt viral propagation, which include ssDNA and ssRNA degradation [11, 18–21], membrane depolarization [12], ATP deamination [13, 22], and NAD^+^ cleavage [40]. By incorporating nucleotide depletion into this broad list of activities, *Bb*Chp and *Ps*Chp expand the range and flexibility of the type III CRISPR–Cas immune response.

## Supplementary Material

gkaf1363_Supplemental_Files

## Data Availability

All data are included in the manuscript or Supplementary data.
