# Supplementary material for: CARF-HAD phosphatase effectors provide immunity during the type III-A CRISPR–Cas response
Source: Nucleic Acids Res. 2025 Dec 19;53(22):gkaf1363. doi: 10.1093/nar/gkaf1363 (PMC12715507; doi:10.1093/nar/gkaf1363)
Supplement: gkaf1363_Supplemental_Files [file gkaf1363_supplemental_files.zip › Supplementary Figures-3.pdf]

SUPPLEMENTARY FIGURES for

CARF-HAD phosphatase effectors provide immunity during the type III-A CRISPR-Cas response

Gianna Stella[1,2] Linzhi Ye[2,3], Sean F. Brady[3], and Luciano Marraffini[1,4,*]

[1] Laboratory of Bacteriology, The Rockefeller University, New York, NY 10065, USA

[2] Tri-Institutional PhD Program in Chemical Biology, Weill Cornell Medical College, Rockefeller University and Memorial Sloan Kettering Cancer Center, New York, NY 10065, USA

[3] Laboratory of Genetically Encoded Small Molecules, The Rockefeller University, New York, NY 10065, US

[4] Howard Hughes Medical Institute, The Rockefeller University, New York, NY 10065, USA

Corresponding author: marraffini@rockefeller.edu

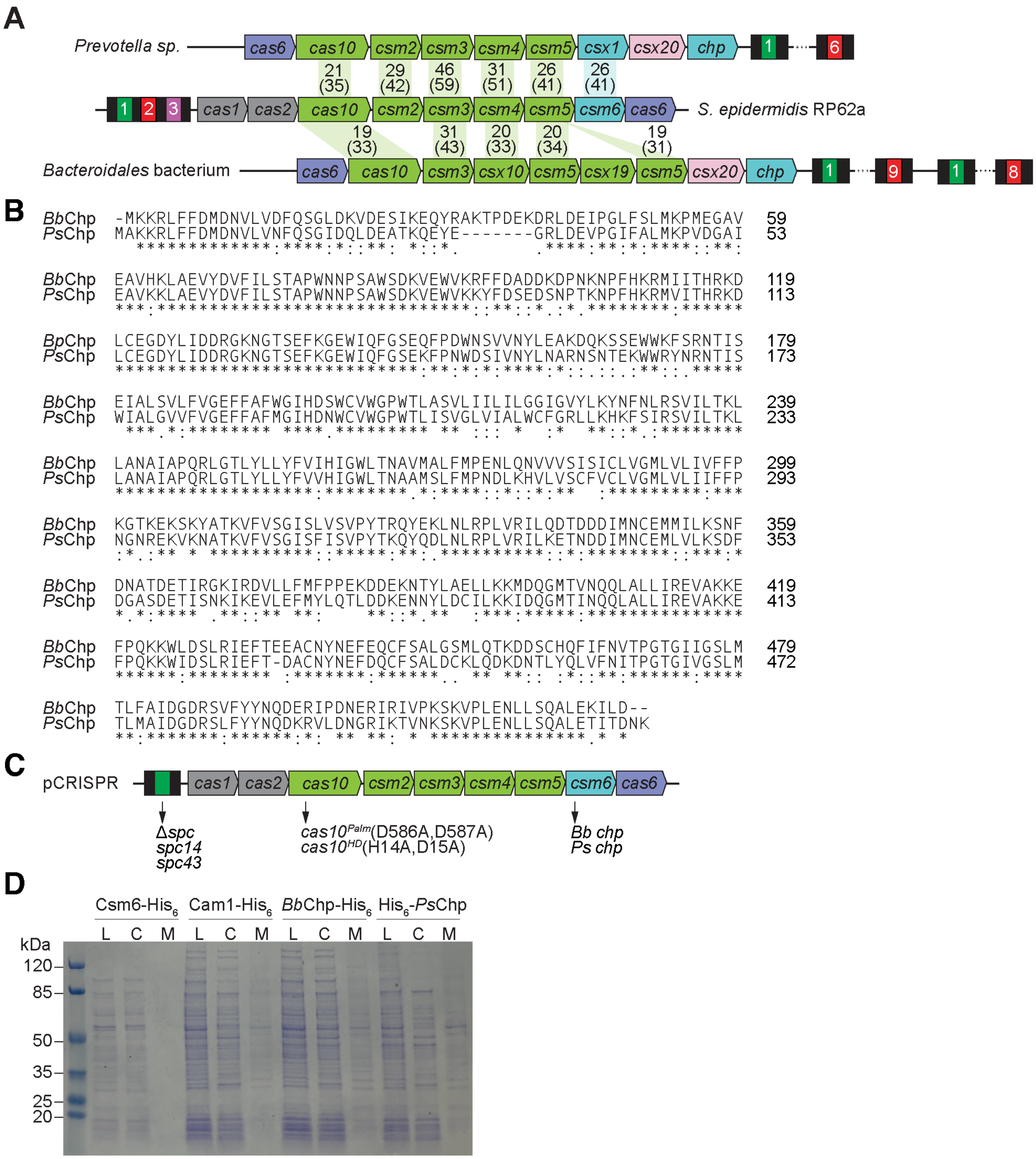

Figure S1. ***Bb*****Chp and** ***Ps*****Chp protein sequences and genetic context.**
(A) Comparison of the III-A systems of *Prevotella sp*. and *Staphylococcus epidermidis* RP62a and the type III-D system of *Bacteroidales* bacterium. Black boxes indicate CRISPR repeats; colored, numbered boxes indicate CRISPR spacers. Numbers indicate the percent homology of amino acid sequences. Numbers in parentheses indicate the percent homology at the DNA sequence level. **(B)** Clustal Omega sequence alignment of full-length *Bb*Chp and full-length *Ps*Chp (*) indicates identical residues; (:)

indicates strongly similar residues; (.) indicates weakly similar residues. **(C)** Genetic modifications of the *S. epidermidis* RP62a type III-A CRISPR locus cloned into various pCRISPR plasmids. Amino acid substitutions and insertion of different spacer sequences are indicated. **(D)** SDS-PAGE with Coomassie staining showing lysate, cytosolic, and membrane fractions used for western blotting experiment in Figure 1E.

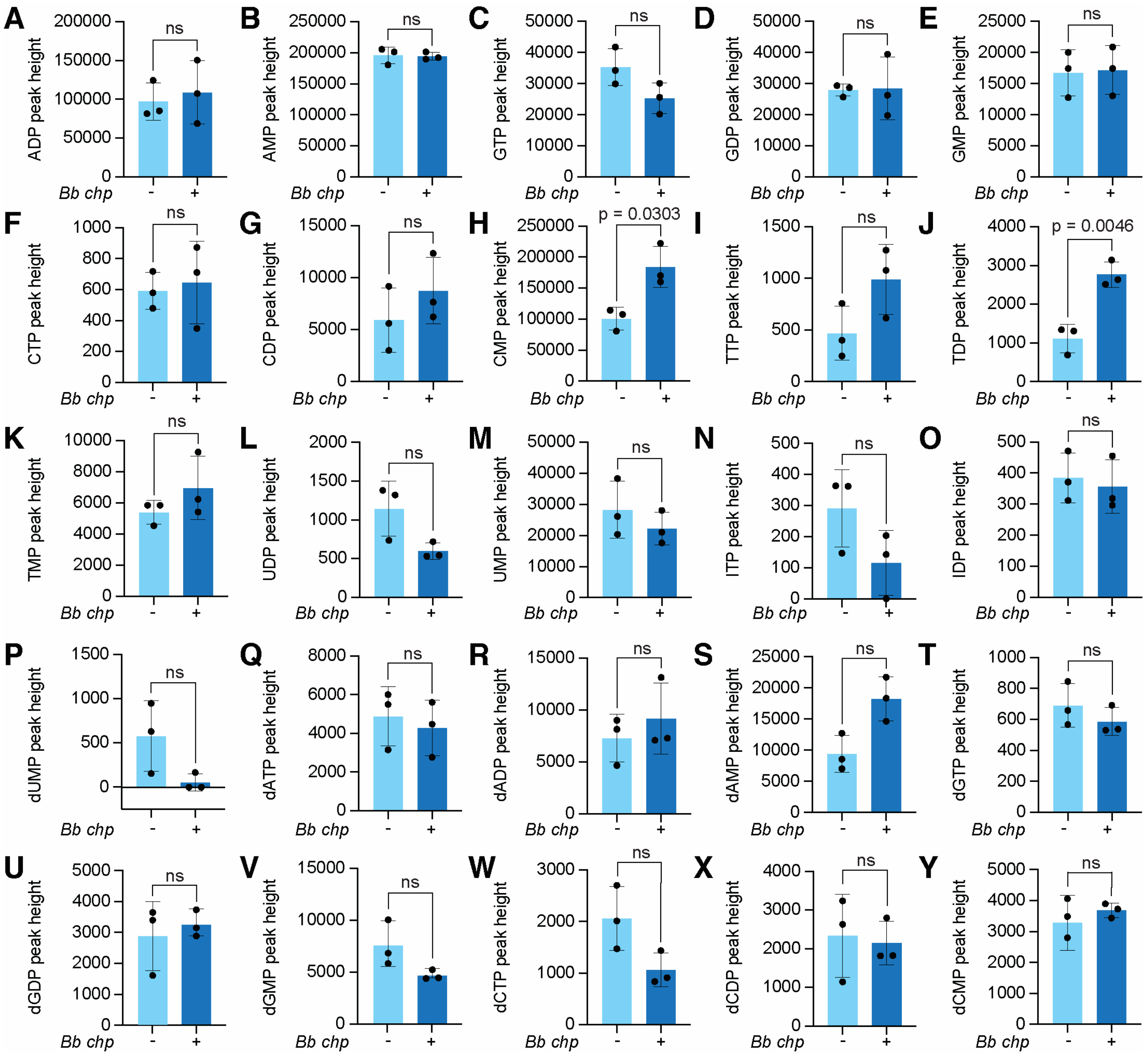

Figure S2. LC/MS analysis of different nucleotides after *Bb*Chp activation in staphylococci.

(A-Y) Quantification of various nucleotide levels from bacterial lysates. Extracts from staphylococci harboring pTarget and pCRISPR(ΔChp) or pCRISPR(*Bb*Chp) were collected after 15 minutes of incubation with aTc and analyzed via LC-MS. Mean of three biological replicates ± SEM is reported. The *p* values shown were obtained with a two-sided *t* test with Welch's correction.

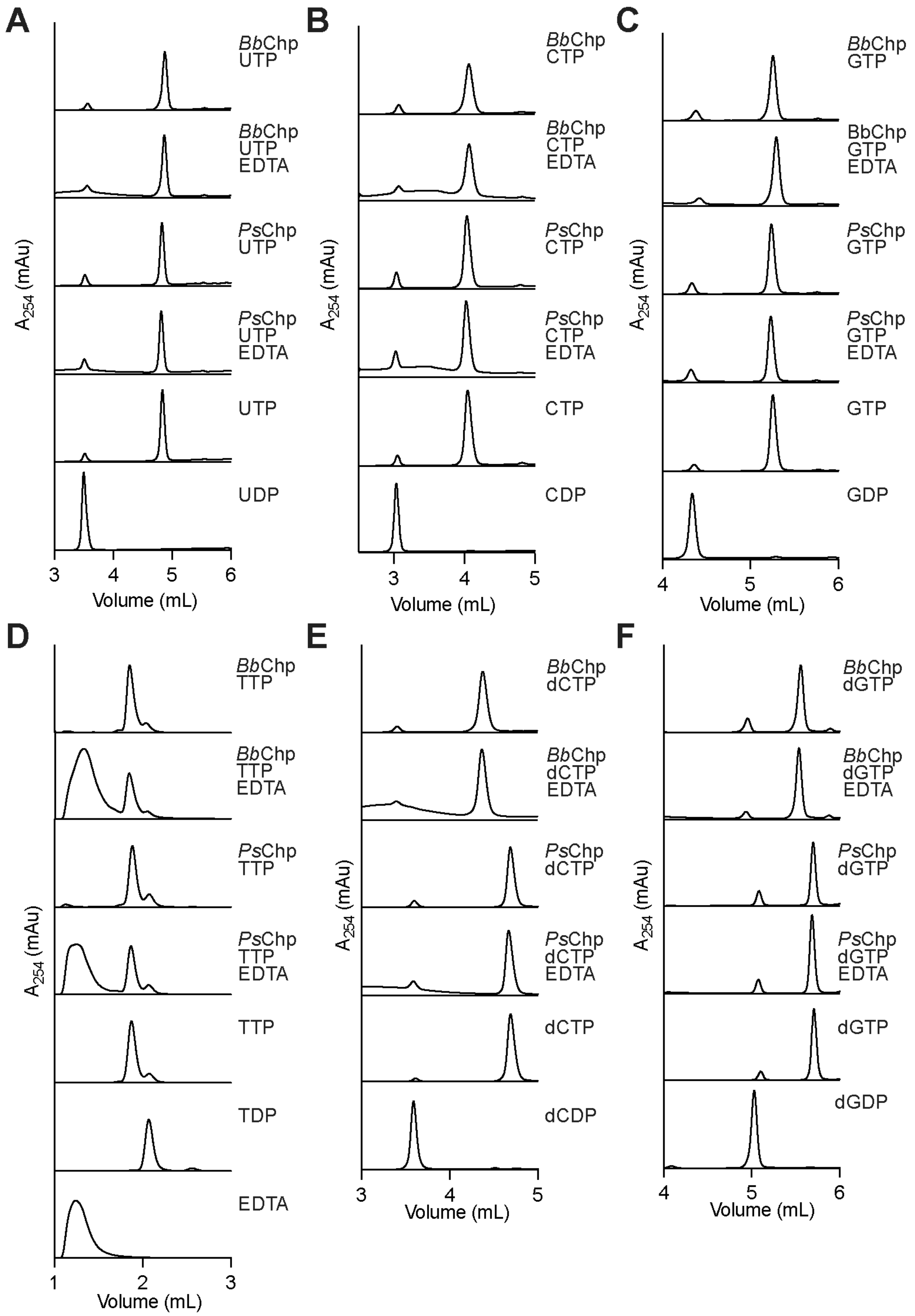

Figure S3. HPLC analysis of *Bb*Chp or *Ps*Chp-HAD treatment of different nucleotides.

(A) HPLC analysis of *Bb*Chp or *Ps*Chp (50 mM) reaction products in the presence UTP (1 mM). Chromatograms of UTP and UDP are shown as standards. Reactions were performed in duplicate. **(B)** Same as **(A)** but in the presence CTP (1 mM). Chromatograms of CTP and CDP are shown as standards. Reactions were performed in duplicate. **(C)** Same as **(A)** but in the presence GTP (1 mM). Chromatograms of GTP

and GDP are shown as standards. Reactions were performed in duplicate. **(D)** Same as **(A)** but in the presence TTP (1 mM). Chromatograms of TTP and TDP are shown as standards. Reactions were performed in duplicate. **(E)** Same as **(A)** but in the presence of dCTP (1 mM). Chromatograms of dCTP and dCDP are shown as standards. Reactions were performed in duplicate. **(F)** Same as **(A)** but in the presence of dGTP (1 mM). Chromatograms of dGTP and dGDP are shown as standards. Reactions were performed in duplicate.

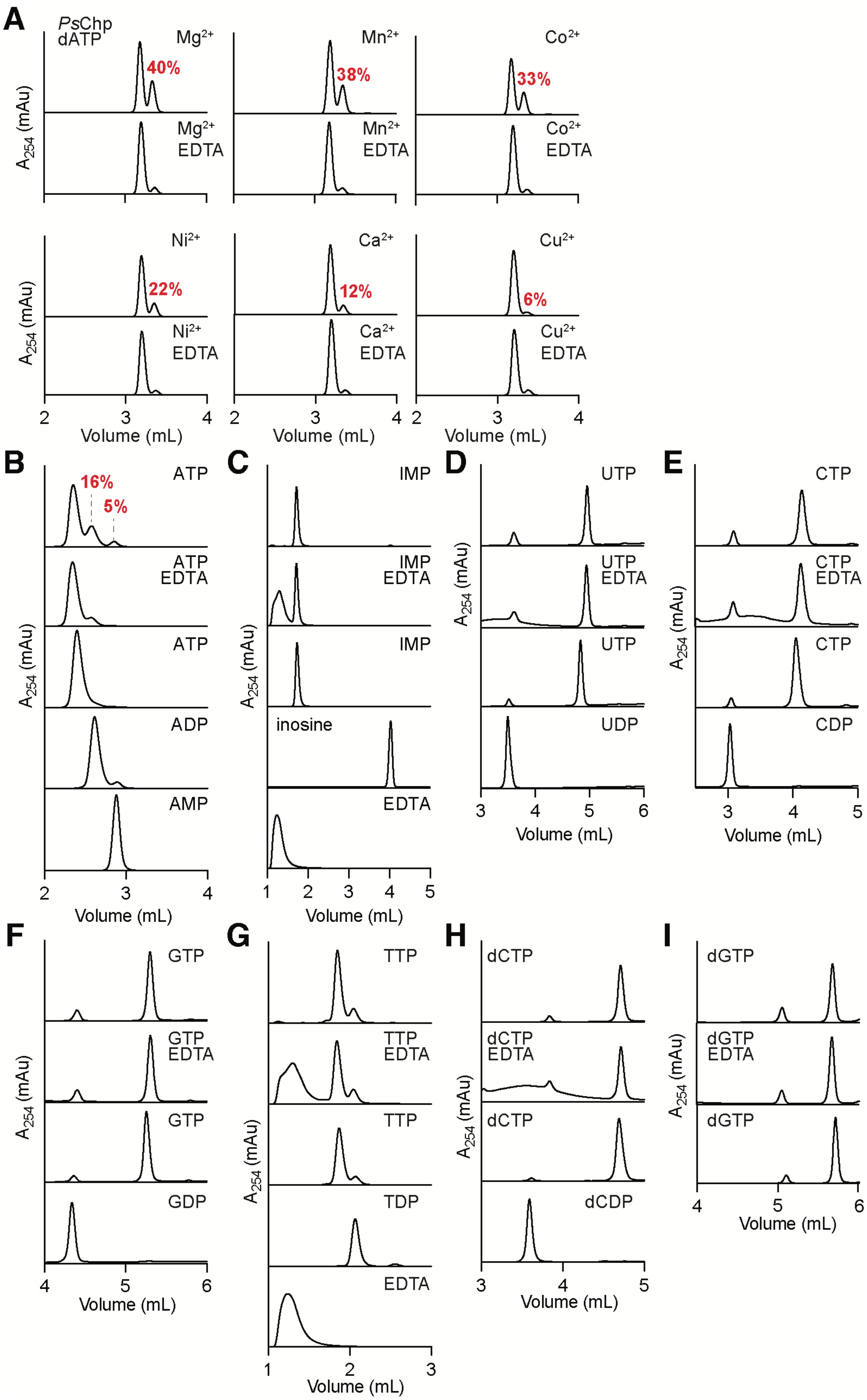
A
PsChp
dATP
Mg2+
40%
Mg2+
EDTA
Mn2+
38%
Mn2+
EDTA
Co2+
33%
Co2+
EDTA
Ni2+
22%
Ni2+
EDTA
Ca2+
12%
Ca2+
EDTA
Cu2+
6%
Cu2+
EDTA
A254 (mAu)
Volume (mL)
B
ATP
16%
5%
ATP
EDTA
ATP
ADP
AMP
C
IMP
IMP
EDTA
IMP
inosine
EDTA
D
UTP
UTP
EDTA
UTP
UDP
E
CTP
CTP
EDTA
CTP
CDP
F
GTP
GTP
EDTA
GTP
GDP
G
TTP
TTP
EDTA
TTP
TDP
EDTA
H
dCTP
dCTP
EDTA
dCTP
dCDP
I
dGTP
dGTP
EDTA
dGTP

Figure S4. HPLC analysis of *Ps*Chp-HAD domain activity with different metals. **(A)** HPLC analysis of *Ps*Chp (50 mM) reaction products in the presence dATP (1 mM) and 1 mM metal Mg^{2+}, Mn^{2+}, Co^{2+}, Ni^{2+}, Ca^{2+}, or Cu^{2+}. Reactions were performed in duplicate. **(B-I)** HPLC analysis of *Ps*Chp HAD domain (50 mM) reaction products in the presence Mg^{2+} (1 mM) and different nucleotides (1mM). **(B)** ATP, **(C)** IMP, **(D)** UTP, **(E)** CTP, **(F)** GTP, **(G)** TTP, **(H)** dCTP, **(I)** dGTP. Reactions were performed in duplicate.

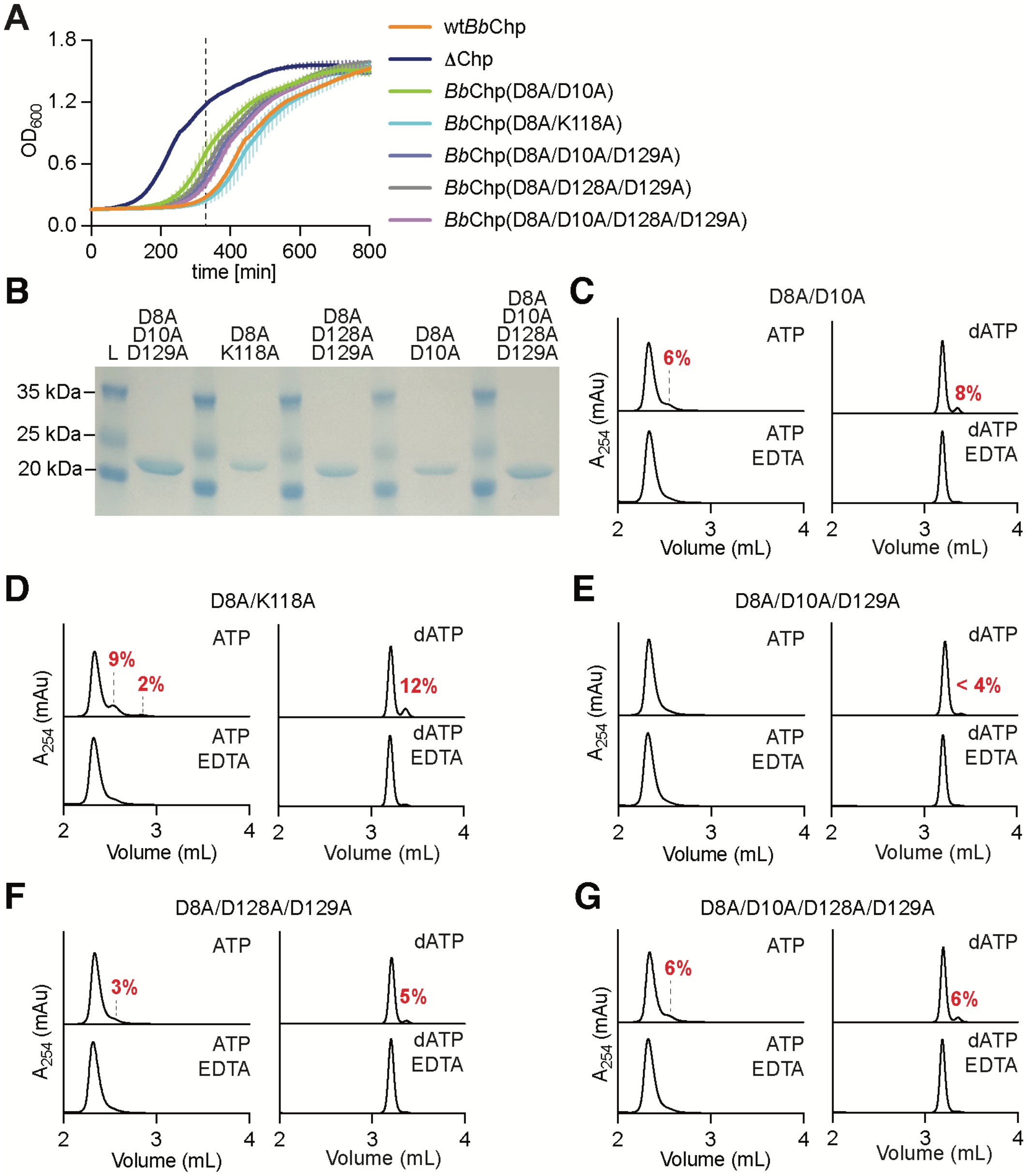

Figure S5. Analysis of putative *Bb*Chp active site residues.
(A) Growth of staphylococci carrying pTarget and pCRISPR dHD with *Bb*Chp variants, measured as OD_{600} after the addition of aTc. Dotted line represents time 330 minutes. Data are mean of two biological triplicates ± SEM. **(B)** SDS-PAGE with Coomassie staining showing purified mutant *Bb*Chp HAD domains. **(C-G)** HPLC analysis of reaction products obtained after treatment of ATP or dATP (1 mM), in the presence or absence of EDTA, with purified mutant *Bb*Chp HAD domains (50 mM). Average percentage of conversion from two duplicates is shown in red.

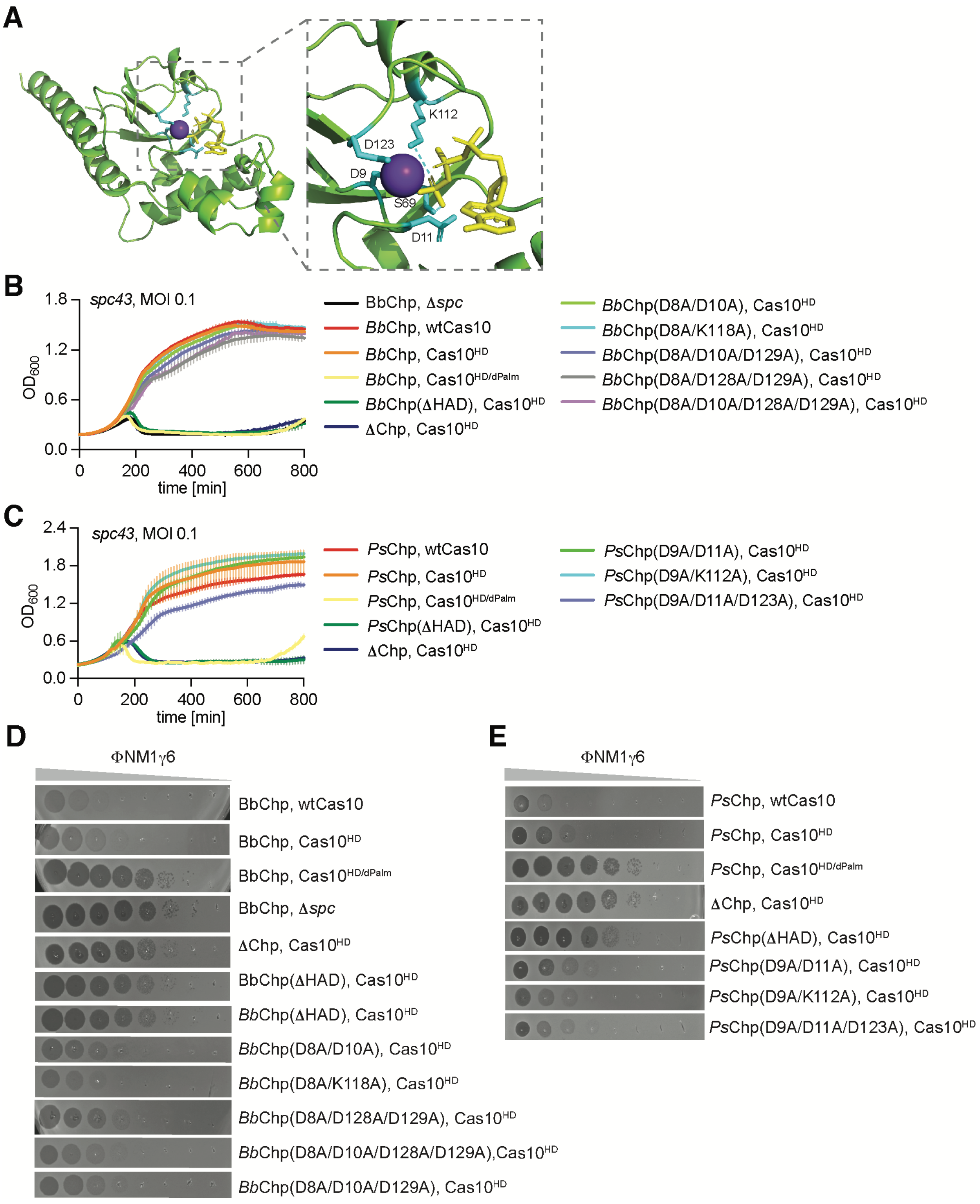

Figure S6. *Bb*Chp and *Ps*Chp mutants provide anti-viral immunity. (A) Alphafold3 structure of HAD phosphatase domain from *Ps*Chp with conserved residues noted. Modeled with ATP and Mn^{2+}. **(B)** Growth of staphylococci carrying pCRISPR with *Bb*Chp variants, measured as OD_{600} after the addition of staphylococcal phage ϕNM1γ6.

(C) Same as **(B)** but with *Ps*Chp variants. **(D)** Plaques formed by phage ϕNM1g6 on *S. aureus* lawns with mutant variants of pCRISPR(*Bb*Chp). **(E)** Same as **(D)** but with *Ps*Chp variants.
